# Immunometabolic Effects of Ginger (*Zingiber officinale* Roscoe) Supplementation in Obesity: A Comprehensive Review

**DOI:** 10.3390/molecules30142933

**Published:** 2025-07-11

**Authors:** María Elizabeth Preciado-Ortiz, Gildardo Gembe-Olivarez, Erika Martínez-López, Juan José Rivera-Valdés

**Affiliations:** 1 Institute of Translational Nutrigenetics and Nutrigenomics, Department of Molecular Biology and Genomics, University Center of Health Sciences, University of Guadalajara, Guadalajara 44340, Jalisco, Mexico; 2 PhD Program in Molecular Biology in Medicine, Department of Molecular Biology and Genomics, University Center of Health Sciences, University of Guadalajara, Guadalajara 44340, Jalisco, Mexico

**Keywords:** immunometabolism, immune cells, gingerol, shogaol, anti-obesity agents, functional foods, chronic diseases

## Abstract

Obesity is a global public health concern characterized by low-grade chronic inflammation and metabolic dysregulation. Ginger (*Zingiber officinale* Roscoe) contains bioactive compounds that have demonstrated potential anti-obesity and immunomodulatory effects. This review aims to synthesize the current evidence regarding the immunometabolic effects of ginger supplementation in obesity, integrating findings from in vitro, in vivo, and clinical studies. Evidence indicates that ginger and its principal compounds, such as 6-gingerol and 6-shogaol, inhibit adipocyte differentiation and lipid accumulation, reduce pro-inflammatory cytokines including tumor necrosis factor alpha (TNF-α), interleukin-6 (IL-6), and the chemoattractant protein of monocytes-1 (MCP-1), improve lipid profiles, and enhance anti-inflammatory adipokines like adiponectin. Clinical trials report improvements in insulin sensitivity, reductions in inflammatory markers, and body weight management in individuals with obesity. This review paper also highlights the cellular and molecular mechanisms of immunometabolic effects of ginger and its bioactive compounds. Therefore, ginger supplementation exhibits promising immunometabolic effects with the potential to support the prevention and treatment of obesity and its comorbidities. However, further rigorous clinical trials are necessary to confirm its efficacy and safety as well as its role in complementing existing strategies for obesity management.

## 1. Introduction

Obesity remains a major global public health challenge, with its prevalence having tripled over recent decades. According to the World Health Organization (WHO), over 2 billion adults are currently overweight, of whom more than 650 million are classified as subjects with obesity, accounting for approximately 13% of the global adult population [1,2,3]. Obesity is characterized by excessive accumulation of adipose tissue, leading to adverse metabolic and physiological consequences. During obesity development, both adipocyte hyperplasia and hypertrophy contribute to profound metabolic and immune dysregulation, promoting endoplasmic reticulum stress, tissue dysfunction, and a state of chronic low-grade inflammation. This inflammatory milieu facilitates the onset of numerous non-communicable diseases, including DM2, cardiovascular disease, and certain types of cancers [2,4,5].

Current strategies for obesity management prioritize dietary modifications and increased physical activity. However, pharmacological interventions are often required in cases where lifestyle changes alone are insufficient. Although anti-obesity drugs offer short-term efficacy, their use is limited by safety concerns, adverse effects, and high costs [5]. Consequently, functional foods and their bioactive compounds have emerged as attractive complementary approaches due to their potential anti-adipogenic, lipolytic, and anti-inflammatory properties [6].

Among these, ginger (*Zingiber officinale*), a widely consumed culinary spice, has garnered significant interest due to its diverse bioactivities, including gastrointestinal protection, anti-cancer potential, and anti-inflammatory and anti-obesity effects [6,7,8,9]. Various studies have independently reported that phenolic constituents of ginger can inhibit adipogenesis, modulate lipid and glucose metabolism, and exert immunomodulatory effects [9,10]. However, the integrated immunometabolic effects of ginger in the context of obesity remain poorly defined and warrant further investigation. Therefore, this review aims to synthesize the current evidence on the immunometabolic effects of ginger supplementation in obesity, with a focus on its cellular and molecular mechanisms, by integrating findings from in vitro, in vivo, and clinical studies.

## 2. Metabolic and Immune Alterations in Obesity

During a state of overfeeding, adipose tissue undergoes expansion in response to increased nutrient availability [11,12]. This adaptive expansion involves both hypertrophy, driven by progressive intracellular accumulation of triglycerides (TG), and hyperplasia, which serves to accommodate excess lipid storage by increasing the number of adipocytes and promoting subcutaneous fat tissue expansion [13,14].

**Figure 1:**
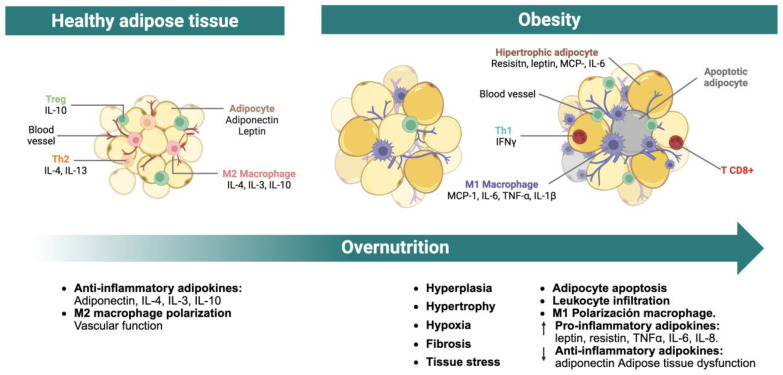
**Hypertrophy of adipose tissue.** This figure illustrates the process of adipose tissue hypertrophy triggered by overnutrition or a positive energy balance. Adipocyte enlargement leads to local hypoxia due to the limited oxygen diffusion relative to the expanded cell size. Hypoxic adipose tissue upregulates profibrotic gene expression, resulting in extracellular matrix remodeling and fibrosis. These changes contribute to adipocyte apoptosis, which in turn promotes immune cell infiltration and a chronic inflammatory state within the tissue. The combined effect of fibrosis, inflammation, and cell death impairs adipose tissue functionality, causing sustained elevations of circulating nutrients such as glucose and lipids. This metabolic dysregulation facilitates ectopic lipid accumulation in peripheral organs, thereby accelerating the development of metabolic diseases. IFNγ, interferon gamma; IL-1β, interleukin-1 beta; IL-3, interleukin-3; IL-4, interleukin-4; IL-6, interleukin-6; IL-10, interleukin-10; IL-13, interleukin-13; MCP-1, Monocyte Chemoattractant Protein-1; T CD8^+^, CD8-positive cytotoxic T lymphocyte; Th1, T helper cell type 1; Th2, T helper cell type 2; TNF-α, tumor necrosis factor alpha; Treg, regulatory T cell.

The adipocyte hypertrophy is strongly associated with the development of local hypoxia and extracellular matrix remodeling, including fibrosis, which sensitizes adipocytes to cellular stress responses [11,15]. Chronic exposure to hypoxic and fibrotic conditions can ultimately trigger adipocyte apoptosis, accompanied by the release of free fatty acids (FFA) and pro-inflammatory mediators. These signals promote immune cell recruitment, particularly macrophages, and stimulate the production of pro-inflammatory cytokines by both leukocytes and adipocytes, perpetuating a state of chronic low-grade inflammation within the tissue microenvironment (Figure 1) [11,13,14,15].

In hypertrophic adipose tissue, the predominant infiltrating immune cells are macrophages and CD8^+^ T lymphocytes. CD8^+^ T cells not only potentiate macrophage recruitment and activation but also promote their polarization toward a pro-inflammatory M1 phenotype in collaboration with adipocytes [11,12,13,14]. M1 macrophages contribute to adipose tissue dysfunction by producing interleukin (IL)-1β, while both macrophages and adipocytes enhance the secretion of pro-inflammatory mediators such as tumor necrosis factor alpha (TNF-α), IL-6, chemoattractant protein of monocytes-1 (MCP-1), and resistin. Simultaneously, adipocyte-derived anti-inflammatory adipokines, particularly adiponectin, are downregulated. These processes create a self-perpetuating cycle of leukocyte recruitment, inflammatory cytokine production, and suppression of anti-inflammatory signals, thereby sustaining chronic low-grade inflammation within the adipose tissue [11,12,16].

The degree of inflammatory mediator accumulation within adipose tissue correlates with the severity of tissue dysfunction [12]. Elevated TNF-α impairs FFA uptake by suppressing fatty acid-binding protein 4 (FABP4, also known as aP2) expression. It also downregulates lipogenic enzymes and perilipins, reducing TG synthesis and storage. Many of these lipogenic genes are regulated by peroxisome proliferator-activated receptor gamma (PPARγ), suggesting that TNF-α may suppress its transcriptional activity [17,18]. Additionally, TNF-α interferes with glucose uptake in peripheral tissues, especially in the muscle, by inhibiting glucose transporter type 4 (GLUT4) translocation to the plasma membrane [18,19].

Equally important, the increased production of IL-6 contributes to adipose tissue dysfunction via multiple mechanisms. Adipocyte-derived IL-6 enhances lipolysis, increasing the release of FFA, which in turn promotes hepatic gluconeogenesis and insulin resistance. IL-6 from M1 macrophages further reinforces the inflammatory microenvironment and suppresses adiponectin expression and secretion [19,20,21].

Similarly, circulating levels of resistin are frequently elevated in obesity and have been implicated in the pathogenesis of insulin resistance. Resistin antagonizes adiponectin signaling and promotes the production of TNF-α and IL-6, as well as adhesion molecules such as intercellular adhesion molecule 1 and vascular cell adhesion molecule 1 [19,20,21]. These effects contribute to a positive feedback loop that sustains the chronic pro-inflammatory and metabolically dysfunctional state of the adipose tissue.

In contrast, adiponectin exerts potent anti-inflammatory and insulin-sensitizing actions. Its circulating levels are inversely correlated with adiposity and are further suppressed by inflammatory cytokines such as IL-6 and TNF-α [11,17]. Adiponectin acts systemically: in the liver, it inhibits gluconeogenesis; in skeletal muscle, it enhances glucose uptake and fatty acid oxidation; and in pancreatic β-cells, it reduces apoptosis and regulates insulin secretion. In both adipose and vascular tissues, adiponectin suppresses the transcription of pro-inflammatory genes mediated by nuclear factor kappa-light-chain-enhancer of activated B cells (NF-κB). It also inhibits lipogenesis induced by sterol regulatory element-binding protein-1 (SREBP-1) and activates PPARα to promote β-oxidation [15,17,18,19]. Therefore, reductions in adiponectin are closely linked to metabolic impairment and systemic inflammation [19].

Leptin, while primarily known for its role in appetite regulation, also influences lipid metabolism by reducing lipogenesis and promoting lipolysis. This action protects non-adipose tissues from lipid overload and supports adipose tissue size reduction [17,18,19]. However, elevated leptin levels in obesity often reflect a state of leptin resistance, compromising its physiological function [11,19].

Collectively, adipocyte hypertrophy, immune cell infiltration, and the imbalance between pro- and anti-inflammatory adipokines converge to maintain a chronic inflammatory milieu within adipose tissue [15]. This inflammatory state disrupts insulin signaling, enhances lipolysis, and impairs lipid storage, resulting in increased circulating FFA and ectopic lipid deposition in peripheral organs, processes that heighten the risk for metabolic diseases [11,13,21].

## 3. Ginger and Its Bioactive Compounds

Ginger (*Zingiber officinale* Roscoe) is a perennial herb belonging to the Zingiberaceae family, originally native to southern Asia [22,23]. The rhizome of ginger, characterized by its distinctive pungent flavor, is one of the most widely consumed culinary spices worldwide [23,24]. Beyond its culinary applications, ginger holds a prominent place in traditional Chinese medicine and is extensively employed in clinical settings for its therapeutic properties [22,23,24].

Ginger root harbors over 300 distinct bioactive constituents, broadly categorized into gingerols, volatile oils, and diarylheptanoids [22]. Gingerols represent the predominant class of bioactive molecules in ginger and are primarily responsible for its characteristic pungency [22,25]. To date, 85 distinct gingerol derivatives have been identified, including gingerols, shogaols, paradols, zingerones, gingerdiones, and gingerdiols. All share a common 3-methoxy-4-hydroxyphenyl functional group, with structural diversity arising from variations in their aliphatic side chains and additional functional groups [22,25]. Among these, 6-gingerol, 6-shogaol, 8-gingerol, and 10-gingerol are the most abundant phenolic compounds in the rhizome [9].

Volatile oils, often referred to as ginger essential oils, confer ginger’s distinctive aroma. A total of 194 volatile oil components have been characterized, predominantly terpenoids, although various alcohols, aldo-ketones, arenes, and fatty hydrocarbons have also been identified [9,22].

Ginger also contains 28 identified diarylheptanoids, a class of phenolic compounds composed of two aromatic rings connected by a seven-carbon aliphatic chain, either linear or cyclic. These molecules exhibit potent antioxidant properties [22,25].

In addition to these primary phytochemicals, ginger root contains a variety of other compounds in lower abundance. These include carbohydrates (e.g., polysaccharides, cellulose, and soluble sugars), a broad spectrum of amino acids (such as glutamate, aspartate, serine, glycine, valine, and tryptophan), organic acids (e.g., citric, succinic, and oxalic acids), and several inorganic elements, including potassium, magnesium, zinc, iron, and manganese [9,22].

**Figure 2:**
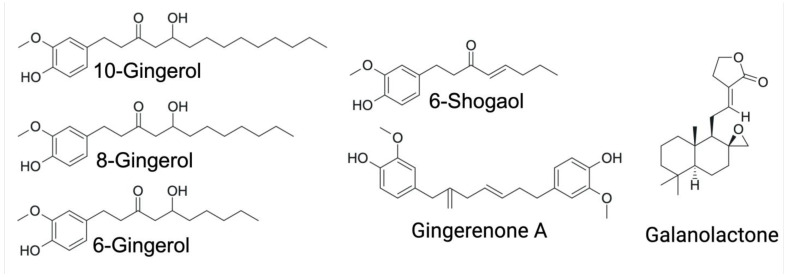
**Representative chemical structures of the major ginger compounds with immunometabolic effects**.

In recent years, substantial research has focused on the pharmacological properties of ginger extracts and their isolated bioactive constituents (Figure 2). These studies have consistently demonstrated that ginger exhibits a broad spectrum of biological activities, including anti-inflammatory, antioxidant, antihypertensive, antiemetic, neuroprotective, and anti-cancer effects, as well as actions that improve glucose metabolism. Notably, recent findings also highlight its potential as an anti-obesogenic agent [6,9,23,25,26,27].

## 4. Immunometabolic Effects of Ginger Bioactive Compounds in Obesity

Immunometabolism refers to an inextricable and bidirectional link between the immune system and metabolism. Since diet plays a crucial role in metabolic homeostasis, it can also affect the status and fate of immune cells, contributing to infectious diseases, inflammation, and obesity. Currently, there is a growing understanding of the intercellular networks for immunometabolism, and the extensive interaction between diet, adipose tissue and the immune system is recognized [28]. Therefore, it is relevant to examine the reported findings on the immunometabolic effects of ginger in obesity (Table 1).

### 4.1. In Vitro Studies

Most in vitro studies have been conducted using 3T3-L1 cells, the preferred cell line for studying the adipogenesis process [29]. The adipogenic differentiation typically lasts 8 days, though this period may be extended depending on the protocols employed.

Various preparations have been tested, ranging from ginger extracts [30,31]. to individual compounds, particularly gingerols and shogaols. Even mixtures of these compounds have been assessed [32], both during the differentiation phase and in adipocytes derived from 3T3-L1 cells.

6-gingerol is the most extensively studied compound. It has demonstrated significant capacity to inhibit adipogenesis and reduce lipid accumulation, with a clear dose-dependent effect [33,34]. Similarly, though less documented, Galanolactone, isolated from Z. officinale, also exhibited inhibitory effects on adipogenic differentiation and lipid droplet accumulation. Doses of 50 and 100 µM of Galanolactone decreased mRNA and protein levels of PPARγ, CCAAT/enhancer-binding protein-alpha (C/EBPα), aP2, and resistin in a dose-dependent manner [35].

In 2017, Suk and collaborators investigated the effects of the five major non-volatile compounds present in ginger individually, all tested at a concentration of 40 μM. Specifically, they evaluated 6-gingerol, 8-gingerol, 10-gingerol, 6-shogaol, and Gingerenone A. Their results indicated that Gingerenone A exerted the strongest anti-adipogenic and anti-lipogenic effects compared to the other compounds [36].

On the other hand, a few studies have examined the impact of ginger compounds on adipocyte hypertrophy. One such case involves 6-shogaol, the dehydrated analog of 6-gingerol, whose anti-adipogenic effects were significantly greater than those of 6-gingerol [37]. In one study, mature 3T3-L1 adipocytes were incubated for 4 h with either 6-shogaol or 6-gingerol at doses of 10, 20, and 40 μM. Only 6-shogaol significantly increased glycerol release and decreased intracellular lipid accumulation, suggesting enhanced lipolysis [37]. In another study, mature 3T3-L1 adipocytes were treated individually with five major ginger compounds at a concentration of 40 μM for four days; Gingerenone A exhibited the strongest inhibitory effect on lipid accumulation in mature adipocytes, with an 18.6% reduction [36]. More recently, the pro-lipolytic effect of 10-gingerol at a dose of 15 µg/mL was demonstrated by an increase in the percentage of glycerol released into the supernatant, accompanied by a 42.16% reduction in lipid accumulation in mature 3T3-L1 adipocytes [38]. Importantly, none of the compounds mentioned above affected the viability of the 3T3-L1 cell line.

Since obesity is characterized by a chronic low-grade inflammatory state, involving sustained activation of the innate immune system [39]. Several in vitro studies have elucidated mechanisms by which ginger compounds regulate the synthesis of inflammatory mediators, modulating cellular activity and the transcriptional regulation of inflammation-associated genes.

In RAW 264.7 macrophage cultures stimulated with lipopolysaccharide (LPS), 6-gingerol administration markedly attenuated reactive oxygen species (ROS) production and inducible nitric oxide synthase (iNOS) expression, indicating interference with canonical inflammatory pathways implicated in obesity pathogenesis [40,41]. Corroboratively, Ho et al. demonstrated that ginger-derived compounds such as 10-gingerol and shogaols downregulated pro-inflammatory gene expression and suppressed interleukin-1 beta (IL-1β) secretion in LPS-activated human macrophages [42]. Furthermore, 8-shogaol was observed to inhibit prostaglandin E2 (PGE2) biosynthesis, a pivotal mediator in chronic inflammatory states, thereby substantiating ginger’s anti-inflammatory potential [43].

Current studies consistently demonstrate the immunometabolic effects of ginger and/or its bioactive compounds. The use of cell-type-specific models has allowed researchers to examine direct effects on metabolic versus immune cells independently. However, it does not fully represent the complexity of the tissue microenvironment or the systemic interplay between metabolic and immune pathways. It is highly recommended to explore co-cultures of adipocytes and immune system cells, as this approach would help confirm the cellular crosstalk involved in orchestrating immunome and metabolic responses and clarify their combined contribution to the effects attributed to them. Furthermore, variations in experimental designs, compound purity, dosage, and bioavailability may limit the reproducibility and translational value of the findings.

### 4.2. In Vivo Studies

Accordingly, preclinical murine models have confirmed the effects of ginger compounds and extracts. Studies have reported that 6-gingerol reduces body weight and improves lipid metabolism [44,45,46,47]. The intake of 0.05% 6-gingerol suppressed body weight gain, white adipose tissue (WAT) mass increase, and adipocyte hypertrophy induced by a high-fat diet (HFD). In addition, 6-gingerol decreased adipose tissue expression of inflammatory adipokines such as TNF-α, MCP-1, plasminogen activator inhibitor-1 (PAI-1), leptin, and resistin. Additionally, it significantly reduced serum insulin, leptin, and TG [45].

Similarly, the daily intraperitoneal administration of 25 mg/kg of 6-gingerol for 4 weeks in C57BL/6J male mice fed with HFD resulted in reduced body weight and significantly suppressed the elevation of total cholesterol (TC) and TG [46]. In Wistar male rats with HFD-induced obesity, 30-day supplementation with 6-gingerol led to lower body weight, reduced adipose tissue, and decreased levels of leptin, glucose, insulin, and serum lipid profiles compared to the control group, particularly at the dose of 75 mg/kg/day [47].

The study by Brahma Naidu, P. et al. [44] included a comparison of 6-gingerol with a reference drug, which is noteworthy as it reinforces the therapeutic context. They evaluated the effects of 6-gingerol oral treatment, at a dose of 75 mg/kg, over 30 days in HFD-induced obese rats and compared the outcomes with a group treated with 10 mg/kg of lorcaserin. Lorcaserin is a selective serotonin 5-HT2c receptor agonist that promotes satiety and reduces caloric intake and was previously used for the treatment of obesity. Gingerol administration resulted in a significant reduction in body weight gain, glucose, and insulin levels, and insulin resistance. It also modified the activity and expression of lipid marker enzymes and inflammatory markers, suggesting that 6-gingerol may serve as an alternative to synthetic drugs in obesity models [44].

Comparable results were observed with a 15-week treatment of 50 mg/kg body weight of Gingerenone A in male C57BL/6J mice fed an HFD. These mice showed lower body weight, reduced visceral fat, smaller adipocyte size, and decreased levels of FFA, although no differences were found in serum or hepatic TG and cholesterol levels compared to the HFD group [36]. While daily consumption of 500 mg/kg body weight of ginger for 16 weeks alleviated HFD-induced increases in body weight, fat accumulation, and serum glucose, TG, and TC levels. It also normalized the HFD-induced alterations in glycolysis and tricarboxylic acid cycle intermediates [48]. Since many in vivo studies use single doses, it is impossible to establish dose–response curves. Furthermore, most lack pharmacokinetic and bioavailability analysis, which limits the interpretation of efficacy results.

Alternative forms of ginger administration have also been explored. One study used ginger water, a white liquid obtained during freeze-drying of fresh ginger rhizomes, to treat Wistar rats for 4 weeks. This intervention reduced body weight gain and improved energy expenditure, in addition to significantly lowering serum TC and TG compared to the control group [49]. However, both the dose and the production and composition of the ginger water were not standardized to allow replication of the observed effects. In addition, ginger extracts have been widely employed in in vivo studies. A steamed ginger extract (SGE) was dissolved in water and administered daily by oral gavage at doses of 40 mg/kg or 80 mg/kg to HFD-fed C57BL/6 mice. SGE reduced body weight gain and fat mass and significantly decreased alanine aminotransferase, aspartate aminotransferase, TC, low-density lipoprotein-cholesterol (LDL-C), and TG levels [31]. Similarly, a hot water extract of ginger also reduced the TG, TC, and LDL-C and increased high-density lipoprotein-cholesterol (HDL-C) in HFD-fed rats [50].

Likewise, the use of a high-hydrostatic pressure extract of ginger (HPG) resulted in 11.5% and 13.4% lower body weight and body weight gain in the HPG-treated groups compared to the HFD group. Serum TG, TC, and LDL-C levels were significantly lower, while HDL-C levels were significantly higher in the HPG group. Additionally, HPG improved hepatic lipid profiles, with reductions in total hepatic lipids, TG, and TC levels. Moreover, HPG had effects on the inhibition of pro-inflammatory cytokine expression in WAT [26].

In this context, the study by Oliveira et al. [51] provides significant evidence of ginger extract’s ability to mitigate metabolic and inflammatory changes induced by refined carbohydrate consumption in a mouse model. Its strengths include the use of a realistic nutritional intervention, focused on a diet rich in simple carbohydrates, which more accurately reflects the modern diet and its adverse effects. They showed that the addition of ginger extract to a high-refined carbohydrate-containing diet in mice leads to lower adiposity, serum cholesterol, TG, and FFA. These metabolic improvements were accompanied by a significant reduction in pro-inflammatory markers in adiposity tissue [51].

Furthermore, supplementation with ginger ethanolic extract significantly reduced HFD-induced weight gain and decreased epididymal fat pad mass, adipocyte size, and visceral fat volume. It significantly lowered serum levels of glucose, insulin, TC, TG, and LDL-C levels while also attenuating the decline in HDL-C induced by HFD [52].

However, it is important to note that the bioactive effects of ginger are closely related to its phytochemical profile, particularly the diversity, type, and concentration of its bioactive compounds. Environmental factors such as geographical origin, soil composition, cultivation conditions, and plant variety have been shown to influence the total phenolic and flavonoid content of ginger, thereby affecting its antioxidant activity [53]. Likewise, the extraction or preparation methods, such as steaming, can lead to variations in the bioactive compounds present and alter the functional activity of herbs or tubers. It has been reported that SGE, compared to a ginger water extract, exhibited polyphenol and flavonoid levels that were 4 and 30 times higher, respectively [52]. Even though it has been suggested, the thermal processing of ginger rhizomes may affect the synthesis of new derivatives with higher biological activity [54]. Therefore, it is necessary that studies report the plant material source and the methodologies used for extraction or preparation of ginger extracts to ensure reproducibility and reliable interpretation of experimental results, particularly in translational and clinical research.

### 4.3. Clinical Trials

Ginger is generally recognized as a safe herb [55]. However, caution is advised in individuals with coagulation disorders or those taking platelet aggregation inhibitors, particularly warfarin. Although earlier reports did not support a clinically relevant bleeding risk [56], recent preclinical evidence has indicated potential antiplatelet effects from the combination of *Caesalpinia sappan* L. and red ginger (*Zingiber officinale* var. *Rubrum*) [57]. Additionally, excessive ginger consumption may disturb the normal homeostasis of cytochrome P450 enzymes (CYPs) and ATP-binding cassette (ABC) transporters, potentially increasing the risk of herb–drug interactions when consumed concomitantly with conventional medications [58]. Nonetheless, the limited clinical evidence, small sample size of the studies, conflicting results, and possible heterogeneity in the contents of herbal products prevent a definitive assessment of these interactions [59].

There are clinical trials in which powdered ginger has been administered, mainly in the form of powdered capsules, to overweight and obese individuals without reporting adverse effects. Doses of 1 g/day did not produce significant changes in anthropometric parameters or body composition in men with obesity, but they did significantly reduce the mean values of C-reactive protein (CRP) levels, indicative of systemic inflammatory mitigation [60]. However, the same dose of 1 g/day of ginger rhizome powder, when administered to children with obesity, led to significant reductions in serum fasting blood sugar, CRP, body mass index (BMI), waist circumference, waist-to-height ratio, alanine aminotransferase, TC, and LDL-C [61]. On the other hand, daily doses of 1600 mg of ginger for 12 weeks were able to reduce TG and TC, although they had no effect on HDL-C and LDL-C. Ginger also significantly lowered fasting plasma glucose, glycated hemoglobin (HbA1c), insulin, CRP, and the homeostatic model assessment of insulin resistance (HOMA-IR) index compared to the placebo group in patients with DM2 [62], a common comorbidity in individuals with obesity.

Ebrahimzadeh Attari V. and collaborators [63] conducted a randomized, double-blind, placebo-controlled study in women with obesity, who received 2 g/day of ginger powder for 12 weeks. Participants in the ginger group showed a significant reduction in BMI, serum insulin, and HOMA-IR index, along with an increase in quantitative insulin sensitivity check index (QUICKI) scores compared to the placebo group [63]. Similar effects were reported by Mozaffari-Khosravi H et al. in patients with DM2 who received 3 g/day of ginger powder for 8 weeks [64]. In longer interventions of 3 months, the daily administration of 3 g of ginger in patients with DM2 who were not receiving insulin significantly improved serum glucose, HbA1c percentage, insulin, insulin resistance, CRP, and total antioxidant capacity [65].

Obesity is a key component of metabolic syndrome (MetS) and, along with insulin resistance, one of the main factors that contribute to the pathogenesis of this disease [66]. Rahimlou M. and colleagues demonstrated that 12 weeks of supplementation with 2 g/day of ginger in patients with MetS significantly reduced fasting blood sugar, serum TG, and insulin resistance [67]. In this regard, ginger supplementation of 3 g per day has shown significant reductions in TC, TG, and LDL-C in patients with hyperlipidemia, supporting the lipid-lowering potential of ginger [68]. This is of clinical relevance, since dyslipidemia, another hallmark of MetS, is closely associated with obesity and non-alcoholic fatty liver disease (NAFLD) [69,70]. Indeed, ginger supplementation has proven effective in the treatment of NAFLD in children with obesity. Thus, ginger may serve as a non-pharmacological intervention to improve liver health in children with obesity, especially when combined with an anti-inflammatory diet, which could further enhance its effects [61]. Similarly, it has been described that combining ginger supplementation with exercise may enhance the benefits on systemic inflammation and metabolic syndrome indices in obese women with breast neoplasms [71]. Another clinical trial involving overweight breast cancer patients showed that ginger capsule supplementation, 3 mg/day for 6 weeks, resulted in a reduction in high-sensitivity CRP, IL-6 and TG. However, the combined intervention with ginger and water-based exercise showed a significantly better effect on pro-inflammatory markers and blood lipids compared to the individual intervention groups. These findings highlight the protective effect of exercise and ginger in the pathogenesis of inflammatory and metabolic responses in overweight women diagnosed with breast cancer [72].

Overall, the sample size of the presented clinical trials is small, limiting the statistical power and generalizability of the results. Furthermore, the longest clinical intervention period was 12 weeks, which prevents the assessment of long-term effects or the potential accumulation of adverse events. Therefore, it is necessary to extend the intervention period and increase the sample size. Equally important is the use of new strategies, such as metabolomics and transcriptomics, which could provide greater clarity and specificity regarding the molecular and cellular mechanisms that may underlie the immunometabolic effects of ginger.

**Table 1:** Key findings on the immunometabolic effects of ginger in obesity.

Clinical Trials					
Author, Year	Subjects	Intervention	Dose	Duration	Results
Kamari N et al. (2023) [61]	160 children with obesity	Ginger rhizome powder	1000 mg/day	12 weeks	Serum fasting blood sugar and high-sensitivity C-reactive protein levels were significantly decreased. Significant reduction in BMI, waist circumference, waist-to-height ratio, ALT, total cholesterol, and LDL-C.
Ebrahimzadeh et al. (2016) [63]	80 obese women (18–45 years)	Ginger powder in tea	2 g/day	12 weeks	BMI, serum insulin, HOMA-IR significantly decreased; QUICKI increased; serum leptin, resistin, and glucose also decreased.
Shidfar F et al. (2015) [65]	45 type 2 diabetic patients	Ginger powder (capsules)	3 g/day	3 months	Improved glucose, HbA1c, insulin, insulin resistance, CRP, antioxidant capacity, PON-1.
Karimi N et al. (2015) [71]	40 women with breast neoplasms	Ginger rhizome powder	4 × 750 mg/day	6 weeks	Reduced IL-10, hs-CRP, insulin; increased HDL-C and HDL/LDL ratio.
Arablou T et al. (2014) [62]	70 type 2 diabetic patients	Ginger rhizome powder	1600 mg/day	12 weeks	Reduced TG, total cholesterol, fasting glucose, HbA1C, insulin, HOMA, CRP, PGE2; no change in HDL or LDL.
Mozaffari-Khosravi H et al. (2014) [64]	88 type 2 diabetic patients	Ginger powder	3 g/day	8 weeks	Reduced fasting blood sugar and HbA1c; improved QUICKI.
Mansour MS et al. (2012) [73]	10 overweight men	Ginger powder (hot water)	2 g	Single dose	Enhanced thermogenesis and reduced hunger and food intake compared to placebo.
Atashak S et al. (2011) [60]	32 obese men	Ginger powder	1 g/day	10 weeks	No effect on lipids or insulin resistance; CRP decreased.
Alizadeh-Navaei R et al. (2008) [68]	85 hyperlipidemic patients	Ginger capsules	3 g/day	45 days	Reduced TG, cholesterol, LDL.
Mohammadzadeh Honarvar et al. (2019) [74]	48 Type 2 diabetic patients	Oral ginger capsules	2 g/day	10 weeks	Trend towards reduced NF-κB; reduced systemic inflammation; decreased hip circumference.
In vivo studies					
Author, Year	Animal Model	Intervention	Dose/Groups	Duration	Results
Hong KH et al. (2023) [45]	Male C57BL/6J mice	6-gingerol	ND, HFD, HFD + 0.05% 6G	8 weeks	Reduced weight gain, WAT mass, adipocyte size, serum insulin/leptin/TG; increased adiponectin; reduced TNF-α, MCP-1.
Cheng Z et al. (2022) [46]	Male C57BL/6J mice	6-gingerol	ND, HFD, HFD + 25 mg/kg	4 weeks	Reduced body weight, TC, and TG levels.
Seo SH et al. (2021) [75]	Male C57BL/6 mice	Ginger powder	-LF: low-fat diet (16% of calories from fat) -HFD -HFD + 5% ginger powder	7 weeks	Reduced body weight, glucose, total cholesterol, and hepatic lipids were observed, along with a reduction in adipocyte size. Additionally, supplementation upregulated the expression of FGF21, ACOX1, CPT1, and antioxidant enzymes SOD1/2, NRF1/2, GPX.
Sayed et al. (2020) [49]	Adult male Wistar rats	Ginger water	Control, 25%, 50% (*v*/*v*)	4 weeks	Ginger water administration significantly reduced serum total cholesterol and triglyceride levels compared to the control group. Treatment with 25% and 50% ginger water induced a 50% and 60% downregulation, respectively, in SREBP-1c mRNA expression, with no changes observed in HSL mRNA levels. In white adipose tissue (WAT), leptin and resistin mRNA expression levels were significantly decreased, whereas adiponectin mRNA expression was upregulated.
Wang J et al. (2019) [48]	Male C57BL/6J mice	Ginger powder	-NCD: normal control diet (10% of calories from fat) -NCD-G: normal control diet supplemented with ginger. -HFD -HFD + G: high-fat diet with ginger supplementation (500 mg/kg, *w*/*w*)	16 weeks	Ginger supplementation alleviated the HFD-induced increases in body weight, fat accumulation, and serum levels of glucose, triglycerides, and cholesterol. It also corrected the HFD-induced alterations in the concentrations of intermediates involved in glycolysis and the TCA cycle.
Kim S et al. (2018) [26]	Sprague–Dawley rats	Ginger Extract	HFD, HFD + WEG/HPG (8 g/kg)	10 weeks	A reduction in body weight, serum lipids (TG, TC, LDL-C), and inflammatory cytokines (TNF-α, IL-6) was observed, along with decreased hepatic levels of total lipids, TG, and TC compared to the HFD group. Additionally, HDL-C levels were significantly higher in the WEG and HPG groups. These effects were associated with AMPK activation and modulation of anti-inflammatory microRNAs, including miR-21 and miR-132, in white adipose tissue (WAT).
Suk et al. (2017) [36]	Male C57BL/6J mice	Gingerol A (GA)	ND, HFD, HFD + GA (10/50 mg/kg)	15 weeks	A reduction in body fat, body weight, adipocyte size, and inflammation was observed, accompanied by increased levels of ATGL and phosphorylated HSL and decreased expression of SREBP-1 and FAS. In addition, macrophage infiltration and the expression of pro-inflammatory markers such as TNF-α and MCP-1 in adipose tissue were reduced. GA also upregulated the expression of genes involved in mitochondrial biogenesis, NRF1 and TFAM, in WAT.
Brahma Naidu et al. (2016) [44]	HFD-induced obese rats	[6]-gingerol	HFD, HFD + 75 mg/kg	30 days	A reduction in body weight, glucose, insulin, and fat was observed. The gingerol-treated group exhibited decreased activity of lipogenic proteins, including HMG-CoA reductase, FAS, PPARγ, and SREBP1c, as well as lower expression of inflammatory markers TNF-α and IL-6 compared to the control group.
Misawa K et al. (2015) [76]	Male C57BL/6J mice	Ginger extract	LF, HFD, HFD + 0.3% GE	18 weeks	Reduced adipocyte size, leptin, cholesterol, insulin, HOMA-R.
Saravanan et al. (2014) [47]	HFD-induced obese rats	[6]-gingerol	HFD + 25/50/75 mg/kg	10 weeks	Supplemented groups exhibited significantly lower body weight and reduced adipose tissue mass, as well as decreased levels of leptin, glucose, insulin, and serum lipid profile parameters.
Oliveira et al. (2018) [51]	In vivo: BALB/c mice on high refined carbohydrate diet	Ginger extract (5% gingerols)	200, 600, 1800 mg/kg	4 weeks	Reduced adiposity, decreased TNF-α and IL-6 (non-significant), and IL-13 in adipose tissue, increased serum adiponectin, and decreased leukocyte infiltration, improving immunometabolic dysfunction.
Kim et al. (2018) [52]	In vivo: Colitis model	Ginger extract	100–500 mg/kg	21 days	Reduced IL-6, TNF-α, and IL-1β in the colon; increased tight junction proteins (ZO-1, occludin), improved epithelial integrity and reduced systemic inflammation.
In vitro studies					
Author, Year	Cell Line	Treatment	Dose	Duration	Results
Tzeng et al. (2013) [33]	3T3-L1 preadipocytes	[6]-gingerol	5, 10, 15 µg/mL	8 days	Inhibited adipogenesis and lipid accumulation; reduced PPARγ, C/EBPα, FAS, aP2; decreased p-GSK3β, Akt.
Li et al. (2015) [34]	3T3-L1 preadipocytes	[6]-gingerol	5, 10, 15 µg/mL	7 days	Adipogenic differentiation was inhibited in a dose-dependent manner, accompanied by reduced mRNA levels and protein expression of PPARγ, C/EBPα, FAS, and ACC. Increased mRNA levels and expression of β-catenin, CCND1, LRP6, and DVL2 were also observed.
Suk et al. (2016) [37]	3T3-L1 preadipocytes	[6]-gingerol, [6]-shogaol	10, 20, 40 µM	6 days	6-Shogaol inhibits adipogenesis and more potently decreases the expression of adipogenic (PPARγ, C/EBPα) and lipogenic (FAS) proteins compared to 6-gingerol.
Rani et al. (2012) [30]	3T3-L1 preadipocytes	Ethyl-acetate ginger extract	0.1–50 µg/mL	10 days	Inhibited differentiation; 50 µg/mL reduced lipids by 43.5%.
Suk et al. (2017) [36]	3T3-L1 preadipocytes	Multiple gingerols and shogaol	40 µM	6 days	Gingerol A showed the strongest anti-adipogenic/lipogenic effect; 10-gingerol was the second most effective.
Ahn et al. (2012) [35]	3T3-L1 preadipocytes	Galanolactone	25, 50, 100 µM	8 days	Dose-dependent inhibition of differentiation and lipid accumulation; decreased the levels of mRNA and proteins PPARγ, C/EBPα, aP2, and resistin.
Lee et al. (2009) [40]	In vitro: RAW 264.7 macrophages stimulated with LPS	6-Gingerol	20, 40, 80 µM	4–12 h	Inhibited iNOS and TNF-α, reduced ROS and intracellular Ca^2+^, blocked PKC-α and NF-κB, showing strong anti-inflammatory and antioxidant effects relevant for obesity.
Ho S. C et al. (2018) [42]	In vitro: Human THP-1 macrophages stimulated with LPS	6-, 8-, 10-Gingerols and Shogaols	5–20 µM	24 h	6-Shogaol inhibited IL-1β secretion by blocking NLRP3 inflammasome activation and reduced TNF-α levels, demonstrating a relevant anti-inflammatory effect in the context of obesity.

ACOX1: Acyl-CoA Oxidase 1, ALT: Alanine Aminotransferase, AMPK: AMP-Activated Protein Kinase, aP2: Adipocyte Protein 2, FABP4: Fatty Acid-Binding Protein 4, ATGL: Adipose Triglyceride Lipase, BMI: Body Mass Index, CCND1: Cyclin D1, C/EBPα: CCAAT/Enhancer Binding Protein Alpha, CPT1: Carnitine Palmitoyltransferase 1, CRP: C-Reactive Protein, DVL2: Disheveled Segment Polarity Protein 2, FGF21: Fibroblast Growth Factor 21, FAS: Fatty Acid Synthase, GPX: Glutathione Peroxidase, HbA1c: Hemoglobin A1c, HDF: High-Fat Diet, HDL-C: High-Density Lipoprotein Cholesterol, HMG-CoA: 3-Hydroxy-3-Methylglutaryl-Coenzyme A, HOMA-IR: Homeostasis Model Assessment of Insulin Resistance, HSL: Hormone-Sensitive Lipase, IL-1β: Interleukin 1 Beta, IL-6: Interleukin 6, IL-13: Interleukin 13, LDL-C: Low-Density Lipoprotein Cholesterol, LF: Low-Fat Diet, LRP6: Low-Density Lipoprotein Receptor-Related Protein 6, MCP-1: Monocyte Chemoattractant Protein 1, NCD: Normal Control Diet, ND: Normal Diet, NF-κB: Nuclear Factor Kappa B, NRF1: Nuclear Respiratory Factor 1, NRF2: Nuclear Factor Erythroid 2-Related Factor 2, PKC-α: Protein Kinase C Alpha, PON-1: Paraoxonase 1, PPARγ: Peroxisome Proliferator-Activated Receptor Gamma, p-HSL: Phosphorylated Hormone Sensitive Lipase, ROS: Reactive Oxygen Species, SOD1/2: Superoxide Dismutase 1 and 2, SREBP-1: Sterol Regulatory Element Binding Protein 1, SREBP-1c: Sterol Regulatory Element-Binding Protein 1c, TC: Total Cholesterol, TFAM: Transcription Factor A, Mitochondrial, TG: Triglycerides, TNF-α: Tumor Necrosis Factor Alpha, WAT: White Adipose Tissue, WEG: Water Extract of Ginger.

## 5. Modulation of Immunometabolic Alterations by Ginger Bioactive Compounds in Obesity

### 5.1. Ginger Compounds Suppress Adipogenesis and Lipogenesis

Several efforts have been undertaken to elucidate the exact mechanisms underlying ginger’s effects on weight loss and metabolic regulation. As a result, multiple potential mechanisms have been proposed, including the suppression of adipogenesis, lipogenesis, and lipid accumulation, as well as the enhancement of lipolysis and thermogenesis in adipose tissue, alongside the regulation and control of appetite [60].

One of the most prominent mechanisms responsible for the anti-obesity action of ginger and its bioactive compounds involves the regulation of gene and protein expression related to adipogenesis and lipogenesis. Key transcription factors such as PPARγ and C/EBPα are well recognized for their pivotal role in triggering adipogenic differentiation [77]. These factors promote the expression of adipocyte-specific genes and proteins, as well as enzymes involved in adipogenesis and the metabolic activities characteristic of adipocytes, including lipogenesis [77,78].

This mechanism has been described for various ginger extracts and bioactive compounds such as Galanolactone (at 50 and 100 µM), which dose-dependently decreased mRNA and protein levels of PPARγ, C/EBPα, aP2, and resistin [35]. Similarly, 6-shogaol and 6-gingerol have shown comparable effects in WAT and in vitro models [31,37]. Tzeng and Liu demonstrated that 6-gingerol (10 and 15 µg/mL) reduced the expression of PPARγ and C/EBPα proteins, consequently lowering the levels of terminal adipogenesis markers such as fatty-acid synthase (FAS) and aP2 [33,46]. Likewise, Li et al. observed inhibited adipogenesis upon treating 3T3-L1 cells with 6-gingerol, showing that this compound induced activation of the Wnt/β-catenin pathway, leading to β-catenin upregulation, which suppresses PPARγ and C/EBPα expression [34].

Furthermore, ginger and its constituents exert anti-lipogenic effects by suppressing the expression and activation of SREBP-1, FAS, and acetyl-CoA carboxylase (ACC). SREBP-1 is a critical lipogenic transcription factor essential for lipid metabolism, as it promotes the expression of genes encoding key enzymes for fatty acid synthesis, including FAS and ACC, in adipocytes [79]. ACC catalyzes the carboxylation of acetyl-CoA to malonyl-CoA, whereas FAS catalyzes the biosynthesis of fatty acids from the two-carbon molecule acetyl-CoA [80].

An in vitro study employing steam-distilled ethanolic ginger extract (SGE) revealed that the reduction in lipid content was accompanied by a significant decrease in mRNA abundance of aP2, GLUT4, FAS, ACC, and adiponectin [81]. Similarly, treatment with 25% and 50% ginger water induced a 50% and 60% reduction in SREBP-1c mRNA expression, respectively [49]. This anti-lipogenic effect has also been reported for 10-gingerol, which was associated with decreased expression of the mechanistic target of rapamycin complex (mTOR) [82]. Since mTOR phosphorylates lipin 1, an inhibitor of SREBP-1, this phosphorylation allows SREBP-1 to translocate to the nucleus and promote transcription of lipogenic genes [83].

In vivo studies with 6-gingerol corroborate reductions in gene and protein expression levels of PPARγ, C/EBPα, SREBP-1, and FAS [45,84]. Supplementation with 6-gingerol in HFD-induced obese rats resulted in decreased activity of lipogenic proteins (HMG-CoA reductase, FAS, PPARγ, and SREBP-1), concomitant with reductions in inflammatory markers such as TNF-α and IL-6 [44]. Consistently, 6-gingerol effectively contributed to the alleviation of adiposity and inflammation in WAT, which has been linked to the regulation of adipokines in diet-induced obese mice [45].

### 5.2. Ginger Compounds Promote Lipid Catabolism

Another crucial mechanism for reducing intracellular lipid droplets in adipocytes is lipolysis, the catabolic process wherein stored TG are hydrolyzed into FFA and glycerol [85]. Compounds such as 6-shogaol and 10-gingerol have demonstrated pro-lipolytic activity, evidenced by increased glycerol release in culture supernatants in vitro [37,38]. This activity was also reported using a mixture of major ginger phenols in 3T3-L1 adipocytes [32]. In vivo supplementation with SGE significantly induced the expression of lipolytic genes, including adipose triglyceride lipase (ATGL) and hormone-sensitive lipase (HSL), in epididymal fat of obese mice [81]. Adipocyte lipolysis is a complex metabolic pathway primarily mediated by ATGL and HSL [86]. Gingerenone A was found to increase ATGL and phosphorylated HSL (p-HSL) at Ser565 expression, along with decreased SREBP-1 and FAS expression compared to HFD-fed mice [36]. Consequently, it has been proposed that ginger’s reduction in hepatic lipid accumulation is related to an enhancement in hepatic fatty acid oxidation [75]. However, in the context of obesity, there is concern that increased lipolytic activity could contribute to the development of hyperlipidemia and/or hepatic steatosis due to elevated release of FFAs into the bloodstream, which promotes TG accumulation in the liver [45]. Nevertheless, ginger supplementation has proven highly effective in improving NAFLD in pediatric patients [61]. Moreover, hepatic TC and TG levels in obese mice were significantly reduced following treatment with ginger extract, which concurrently downregulated hepatic genes involved in lipid metabolism, such as FAS and SREBP-1 [52]. Similar reductions in hepatic lipid content have been reported in Sprague Dawley rats subjected to HFD and treated with various ginger extracts [26].

The reduction in liver fat following ginger supplementation can also be attributed to enhanced fatty acid oxidation in hepatocytes. FFA are metabolized to produce energy through mitochondrial β-oxidation [87,88]. Compounds such as 6-gingerol, 10-gingerol, and ginger powder have been shown to modulate the expression of enzymes critical for lipid metabolism, including FAS, ACC, and carnitine palmitoyltransferase-1 (CPT-1) [38,44,75]. CPT-1 is the key enzyme governing the initiation and regulation of fatty acid oxidation within mitochondria [89]. Hepatic lipid accumulation was significantly diminished and associated with upregulation of fibroblast growth factor 21 (FGF21), acyl-CoA oxidase 1 (ACOX1), and CPT1, along with decreased FAS in HFD mice supplemented with ginger powder [75]. Furthermore, Gingerenone A enhanced the expression of mitochondrial biogenesis-related genes such as nuclear respiratory factor 1 (NRF1) and mitochondrial transcription factor A (TFAM) in epididymal WAT through AMP-activated protein kinase (AMPK) activation [36]. Another study implicated AMPK-SIRT1-mediated mitochondrial biogenesis and thermogenesis as mechanisms linked to SGE in obesity models [31]. SGE also increased the uncoupling protein one (UCP1) expression, which is expressed in the mitochondria and is strongly associated with the browning and thermogenesis of adipose tissue [90]. Increasing thermogenesis, through the synthesis and expression of UCP1, is one known mechanism by which ginger promotes energy expenditure. It has been suggested that ginger compounds may activate the sympathetic nervous system and the release of norepinephrine (NE) through the transient receptor potential vanilloid type 1 (TRPV1), leading to an increase in UCP1 expression and thermogenesis in adipose tissue [6].

### 5.3. AMPK: A Key Molecule in the Immunometabolic Effects of Ginger

AMPK is a principal regulator of cellular energy homeostasis, orchestrating anabolic and catabolic pathways [91]. The administration of ginger extract in HFD mouse models demonstrated that both mTOR and SREBP expression were modulated via AMPK pathway activation [31]. Activation of AMPK attenuates lipogenesis and TG synthesis while promoting lipolytic pathways and fatty acid oxidation [26,31]. Kim et al. [26] reported up to a 1.8-fold increase in AMPK activity in the ginger extract-supplemented group compared to HFD controls, which influenced the regulation of genes associated with adipogenesis and inflammation in WAT. Notably, AMPK exerts immunomodulatory effects by preventing the expression and secretion of pro-inflammatory cytokines [26].

Activation of AMPK plays a fundamental role in the modulation of inflammation, particularly in the context of obesity. Several bioactive compounds present in ginger, such as 6-gingerol, exert immunomodulatory effects via AMPK activation. This AMPK activation has been shown to negatively regulate pro-inflammatory cytokines, including TNF-α, IL-6, and MCP-1, which are key mediators of chronic inflammation associated with obesity [26]. Similarly, Hong et al. (2023) demonstrated that supplementation with 6-gingerol in an in vivo model attenuated inflammation in WAT through AMPK activation, which inhibited inflammatory pathways by reducing the expression of TNF-α and MCP-1, while promoting the production of adiponectin, an anti-inflammatory adipokine [45].

These effects are complemented by recent findings showing that the administration of ginger extract not only decreases classical pro-inflammatory cytokines but also modulates the expression of key adipokines involved in adipose tissue metabolism and inflammation. Treatment with ginger water significantly reduced leptin mRNA expression, a pro-inflammatory adipokine implicated not only in body weight regulation but also in inflammatory processes and insulin resistance [49]. In hyperleptinemic states, leptin receptor (LEPR) resistance may develop, impairing normal intracellular leptin signaling [92]. Concurrently, adiponectin expression, known for its anti-inflammatory properties and ability to enhance insulin sensitivity, was increased, whereas resistin expression, an adipokine linked to insulin resistance and inflammatory processes, was suppressed. These alterations in leptin, adiponectin, and resistin contribute to improved metabolic profiles and reduced chronic inflammation related to obesity [49]. These data align with in vitro studies reporting a significant decrease in resistin levels in 3T3-L1 cells following treatment with Galanolactone [35]. Likewise, 10-gingerol has been reported to increase adiponectin and leptin concentrations while decreasing resistin and TNF-α levels [93]. Complementary in vivo studies have documented reduced plasma leptin concentrations [47]. Collectively, these findings suggest AMPK as a pivotal regulator of inflammation, particularly through its capacity to inhibit pro-inflammatory cytokines. Since the administration of ginger extract correlates with elevated serum adiponectin levels, contributing to the downregulation of TNF-α, IL-6, and IL-13 expression, thereby exerting anti-inflammatory effects [51].

TNF-α is a pro-inflammatory cytokine that induces cellular activation and contributes to both systemic and localized inflammation [94]. Meanwhile, IL-13 can induce fibrosis in adipose tissue through activation of the JAK/STAT6 signaling pathway, stimulating the expression of extracellular matrix-related genes and disrupting the tissue’s functional architecture [95]. Consistently, Kim et al. (2018) reported that ginger extract significantly reduced TNF-α, IL-6, and MCP-1 in rats fed a HFD [26]. MCP-1 is a chemokine that promotes the recruitment of monocytes, which differentiate into pro-inflammatory macrophages, thus sustaining chronic inflammation. MCP-1 expression is elevated under insulin-resistant conditions, favoring its sustained expression [96,97]. Furthermore, ginger extract has been shown to reduce macrophage infiltration in adipose tissue and significantly downregulate inflammatory markers such as TNF-α, IL-6, and MCP-1, correlating with improved inflammatory and metabolic states [45,76].

The interplay between AMPK activation and inflammation inhibition may be mediated by suppression of NF-κB, a key transcription factor in inflammatory responses that regulates genes such as TNF-α and MCP-1. NF-κB activation is crucial for immune responses by facilitating recruitment of immune cells like macrophages, which perpetuate inflammation [41]. The inhibition of NF-κB by AMPK has been demonstrated in multiple studies, including those by Oliveira et al., 2018, Suk et al., 2017, and Lee et al., 2009 [36,40,51], which observed that 6-gingerol-induced AMPK activation primarily attenuated inflammation through NF-κB inhibition. NF-κB suppression is associated with reduced ROS production and iNOS expression, two critical mediators of inflammation. Lee et al. (2009) [40] demonstrated that 6-gingerol inhibits ROS production and iNOS expression in LPS-stimulated macrophages by blocking protein kinase C-alpha (PKC-α) and NF-κB signaling pathways. PKC-α activation increases ROS generation, which in turn activates NF-κB, triggering the production of pro-inflammatory cytokines and other inflammatory mediators. In this context, 6-gingerol disrupts this inflammatory cycle by inhibiting PKC-α, thereby attenuating the inflammatory response [40].

The impact of AMPK activation and NF-κB inhibition is not confined to in vitro or animal models. In a clinical study involving patients with metabolic diseases such as DM2, supplementation with 6-gingerol reduced NF-κB concentrations. This reduction suggests that AMPK activation by ginger may mitigate systemic inflammation mediated by ROS and NF-κB, a mechanism particularly relevant to metabolic disorders like diabetes [74].

### 5.4. Ginger Exhibits Epigenetic Mechanisms to Modulate Inflammation

Epigenetic mechanisms through which ginger modulates inflammation have also been described, involving the regulation of microRNA (miRNA) expression, particularly miR-21 and miR-132. miRNAs are small non-coding RNA molecules, approximately 18 to 25 nucleotides in length, that regulate gene expression post-transcriptionally by binding to complementary sequences on target messenger RNAs, leading to translational repression or degradation [98]. According to Kim Seunghae et al. (2018), these miRNAs are involved in inflammation and adipogenesis [26]. Specifically, miR-21 promotes adipogenesis by directly inhibiting the TGF-β receptor 2 (TGFBR2), thereby reducing TGF-β signaling, which normally suppresses adipocyte differentiation [99]. Furthermore, miR-21 is known to regulate inflammation by modulating the expression of various cytokines and immune-related genes, suppressing targets such as programmed cell death protein 4 (PDCD4) and phosphatase and tensin (PTEN), thereby facilitating IL-10 production and regulating TNF-α levels [100,101]. Conversely, miR-132 plays a critical role in NF-κB activation by promoting the expression of inflammatory genes such as TNF-α and IL-1β through direct inhibition of SIRT1, a protein that normally restrains this pathway; this enables sustained NF-κB activity and enhanced transcription of pro-inflammatory cytokines [102]. Treatment with 6-gingerol reduced expression levels of miR-21 and miR-132 in WAT, contributing to decreased inflammation [26,102].

### 5.5. Ginger Compounds Reduce Chronic Inflammation

Moreover, ginger constituents such as 6-, 8-, and 10-gingerols and shogaols have been shown to reduce the active form of pro-inflammatory cytokines like IL-1β. In vitro studies demonstrated that 6-gingerol inhibits the expression of NLR Family Pyrin Domain Containing 3 (NLRP3), a key component of the inflammasome responsible for recruiting caspase-1 to cleave pro-IL-1β into its active form, IL-1β. By blocking NLRP3, ginger compounds impede this conversion, thereby interfering at multiple points within the inflammatory pathway and diminishing IL-1β production [42]. Additionally, compounds such as 8-shogaol have been investigated in aged human myoblasts, where they reduced prostaglandin biosynthesis, including PGE2, a well-known mediator of chronic inflammation. PGE2 is a lipid molecule that promotes inflammation and pain through activation of specific cell surface receptors, such as EP1 and EP2. Elevated PGE2 levels in inflammatory diseases like obesity contribute to immune dysregulation. Ginger’s capacity to reduce PGE2 biosynthesis aids in modulating chronic inflammation associated with aging and metabolic diseases [43].

### 5.6. Ginger Modulates Microbiota Composition and Function

Ginger has been shown to positively influence the composition and functionality of the gut microbiota in both experimental and clinical models. In a study conducted in mice fed a HFD, ginger supplementation at a dose of 500 mg/kg of body weight significantly reduced body weight, hepatic steatosis, and insulin resistance. These effects were associated with modulation of the gut microbiota, including an increase in beneficial bacteria such as *Bifidobacterium*, *Alloprevotella*, and *Allobaculum,* which are known short-chain fatty acid (SCFA) producers, suggesting that these microbial changes may contribute to the metabolic benefits of ginger. *Bifidobacterium* is a common member of the healthy gut microbiota, and its imbalance in both number and composition is a frequent alteration in various diseases [103].

Similarly, 6-gingerol, in addition to reducing body weight and improving lipid profiles, led to a remodeling of the gut microbiota, with increased abundance of genera such as Muribaculaceae, *Alloprevotella*, and *Akkermansia*. To determine whether these effects were mediated by microbiota, a fecal microbiota transplant was performed in mice. The results showed that recipients of microbiota from 6-gingerol-treated donors also exhibited reduced weight gain and a similar microbial composition, supporting the critical role of these microorganisms in the observed effects [104].

Moreover, clinical studies in healthy humans have reported that daily consumption of fresh ginger juice for one week led to a significant increase in the number of intestinal bacterial species, an increase in *Faecalibacterium,* a genus with anti-inflammatory properties, and a trend toward an elevated *Firmicutes/Bacteroidetes* ratio, along with a decrease in the *Prevotella/Bacteroides* ratio and in pro-inflammatory genera such as *Ruminococcus_1* and *Ruminococcus_2*. These genera harbor genes associated with oxidative stress response, adhesion, and mucus degradation, which facilitates their adaptation to an inflamed environment [105,106].

Additionally, a double-blind clinical trial in healthy adults who consumed 1.2 g/day of ginger powder for 14 days showed an increased relative abundance of the phylum Actinobacteria, as well as *Parabacteroides*, *Bacillus*, and *Ruminococcaceae incertae sedis* bacteria associated with immune, antimicrobial, and digestive functions. These findings support the substantial impact of ginger on the diversity and functional structure of the gut microbiota, which in turn influences host metabolism and inflammation [107].

### 5.7. Ginger Improves Hepatic and Serum Lipid Profiles

In addition, clinical and preclinical evidence suggests that ginger supplementation results in weight loss accompanied by improvements in metabolic serum markers such as blood glucose and lipid profiles [36,45,46,48,61,65,75].

In this context, 6-gingerol significantly reduced serum TG, FFA, TC, very-low-density lipoprotein (VLDL), and LDL-C levels, while increasing HDL-C in HFD-induced obese rats [47]. Moreover, 6-gingerol suppressed HFD-induced body weight gain, WAT mass increase, and adipocyte hypertrophy, accompanied by reductions in serum insulin, leptin, and TG levels, alongside a significant increase in serum adiponectin levels [45].

Ginger has also demonstrated a hypotriglyceridemic effect, potentially mediated through the stimulation of lipoprotein lipase (LPL) activity, which facilitates the hydrolysis of circulating TG and consequently lowers serum TG levels [62,108]. Additionally, 6-gingerol prevents HFD-induced hyperlipidemia by modulating the expression of enzymes critical to cholesterol metabolism, including lecithin-cholesterol acyltransferase (LCAT) and LPL, as well as inflammatory markers such as TNF-α and IL-6 [44].

Among the mechanisms by which ginger contributes to cholesterol reduction is the inhibition of its biosynthesis, as reported in the livers of rats treated with ginger compounds [31,48,68,75]. Another mechanism involves the increased conversion of cholesterol into bile acids, given that ginger enhances hepatic cholesterol-7α-hydroxylase activity, resulting in decreased serum cholesterol levels [61,68]. Furthermore, ginger has been proposed to increase fecal excretion of cholesterol and phospholipids, thereby reducing circulating cholesterol levels [62,109].

### 5.8. Ginger Improves Glycemic Control and Enhances Insulin Sensitivity

Complementary to improvements in both hepatic and serum lipid profiles, ginger supplementation has been shown to reduce fasting plasma glucose, HbA1c, insulin levels, and the HOMA-IR index [48,62,65,75].

Ginger may contribute to glycemic control by inhibiting hepatic phosphorylase, an enzyme responsible for glycogen degradation in hepatocytes, thereby compromising the mobilization of glucose reserves. In addition, ginger inhibits glucose-6-phosphatase activity, reducing the conversion of glucose-6-phosphate into glucose and thus lowering blood glucose levels [110,111]. Within the gastrointestinal tract, ginger may interfere with carbohydrate digestion by inhibiting α-glucosidase and amylase enzymes, leading to reduced glucose absorption [47,61,112].

Moreover, ginger appears to modulate lipid and glucose metabolism by downregulating hepatic expression of the carbohydrate-responsive element-binding protein (ChREBP) gene. ChREBP, activated by glucose, promotes glycolysis and lipogenesis in the liver and induces de novo lipogenesis in adipose tissue through regulation of key enzymes such as ACC, FAS, and glucose-6-phosphatase [113]. Inhibition of ChREBP results in decreased lipogenic and gluconeogenic activities, reduced hepatic fat accumulation and serum TG levels, and improved insulin resistance [62,111].

Insulin resistance (IR) occurs when cells inadequately respond to insulin, disrupting blood glucose homeostasis and increasing the risk of DM2 [96,114]. IR activates lipolysis in adipose tissue, increasing FFA delivery to the liver, which contributes to steatosis [96]. Fortunately, ginger treatment significantly reduced hepatic steatosis in clinical trials [61].

One putative mechanism by which ginger enhances insulin sensitivity is via upregulation of adiponectin, an adipokine whose serum levels are typically reduced in obesity and insulin resistance. Both 6-gingerol and ginger water extracts have been shown to increase circulating adiponectin levels [45,49,52]. Since adiponectin expression is regulated by PPARγ, activation of this transcription factor or the agonistic activity of ginger compounds such as 6-shogaol may also lead to increased adiponectin and consequent improvements in insulin sensitivity [110,115]. Furthermore, ginger’s properties may enhance insulin sensitivity by upregulating GLUT4, increasing insulin receptor expression, and supporting pancreatic β-cell function, ultimately promoting better glucose tolerance [62,112].

**Figure 3:**
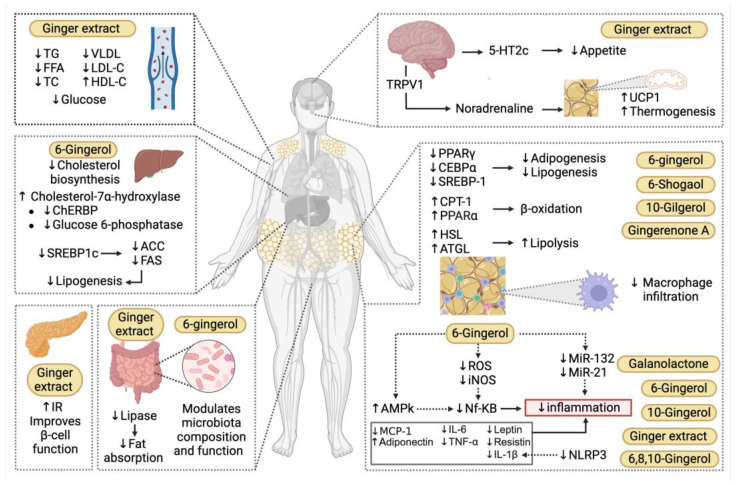
**Mechanisms underlying the immunometabolic effects of ginger in obesity**. Ginger and its bioactive compounds exert immunomodulatory effects across multiple organs. Among their principal actions is the capacity to suppress adipogenesis and lipogenesis by regulating key transcription factors such as Peroxisome Proliferator-Activated Receptor Gamma (PPARγ), CCAAT/Enhancer-Binding Protein Alpha (CEBPα), and Sterol Regulatory Element-Binding Protein 1 (SREBP-1), thereby limiting the formation of new adipocytes and lipid storage. Additionally, ginger promotes lipid catabolism by stimulating lipolysis through the activation of enzymes such as hormone-sensitive lipase (HSL) and triglyceride lipase (ATGL), while also enhancing mitochondrial β-oxidation of fatty acids via carnitine palmitoyltransferase-1 (CPT-1). In the central nervous system, ginger influences appetite regulation through the 5-Hydroxytryptamine Receptor 2C (5-HT2c) pathway and also activates the sympathetic nervous system, leading to norepinephrine release that stimulates thermogenesis in brown adipose tissue via activation of Uncoupling Protein 1 (UCP1). Ginger and its constituents further activate the AMP-Activated Protein Kinase (AMPK) pathway, which results in inhibition of the transcription factor NF-κB, thereby reducing the expression of pro-inflammatory cytokines such as Interleukin-6 (IL-6), Tumor Necrosis Factor Alpha (TNF-α), and Interleukin-1 Beta (IL-1β), as well as decreasing macrophage infiltration in adipose tissue by downregulating Monocyte Chemoattractant Protein-1 (MCP-1). Moreover, ginger has been shown to modulate inflammation via epigenetic mechanisms, including the downregulation of pro-inflammatory microRNAs such as miR-132 and miR-21. In the intestine, ginger extract decreases fat absorption by reducing lipase activity. Ginger modulates the composition and function of the intestinal microbiota, which in turn influences host metabolism and inflammation. In the pancreas, ginger supports improved β-cell function and insulin sensitivity. In the liver, compounds such as 6-gingerol contribute to the improvement of lipid profiles both in hepatic tissue and circulation by inhibiting lipogenesis through suppression of SREBP-1, Acetyl-CoA Carboxylase (ACC), and Fatty Acid Synthase (FAS), and by promoting the expression of cholesterol-7α-hydroxylase, thereby reducing cholesterol biosynthesis. Positive effects on the serum lipid profile have also been observed, including reductions in triglycerides (TG), free fatty acids (FFA), total cholesterol (TC), Very-Low-Density Lipoprotein (VLDL), and Low-Density Lipoprotein Cholesterol (LDL-C), alongside an increase in High-Density Lipoprotein Cholesterol (HDL-C) and a decrease in blood glucose levels 5-HT2c, 5-Hydroxytryptamine Receptor 2C; ACC, Acetyl-CoA Carboxylase; ACTL, triglyceride lipase; AMPK, AMP-Activated Protein Kinase; CEBPα, CCAAT/Enhancer-Binding Protein Alpha; ChREBP, Carbohydrate-Responsive Element-Binding Protein; CPT-1, Carnitine Palmitoyltransferase-1; FAS, Fatty Acid Synthase; FFA, Free Fatty Acids; HDL-C, High-Density Lipoprotein Cholesterol; HSL, hormone-sensitive lipase; IL-1β, Interleukin-1 Beta; IL-6, Interleukin-6; iNOS, Inducible Nitric Oxide Synthase; IR, Insulin Receptor; LDL-C, Low-Density Lipoprotein Cholesterol; miR-21, MicroRNA-21; miR-132, MicroRNA-132; NF-κB, Nuclear Factor Kappa B; NLRP3, NOD-LRR- and Pyrin Domain-Containing Protein 3; PPARα, Peroxisome Proliferator-Activated Receptor Alpha; PPARγ, Peroxisome Proliferator-Activated Receptor Gamma; ROS, Reactive Oxygen Species; SREBP1c, Sterol Regulatory Element-Binding Protein 1c; TC, Total Cholesterol; TG, Triglycerides; TNF-α, Tumor Necrosis Factor Alpha; TRPV1, Transient Receptor Potential Vanilloid 1; UCP1, Uncoupling Protein 1; VLDL, Very-Low-Density Lipoprotein.

In this regard, HbA1c is a standard diagnostic marker for evaluating long-term glycemic control and risk of diabetic complications that reflects average blood glucose over the prior three months [116]. Meta-analyses have concluded that dietary ginger significantly improves HbA1c from baseline to follow-up, suggesting a sustained impact on glycemic control in patients with DM2 [117]. Therefore, ginger can be considered an effective adjunct in the prevention and management of diabetes and its complications (Figure 3).

## 6. Conclusions

Adipose tissue is a reservoir for numerous immune cells, which act together to regulate systemic metabolism and modulate inflammation within the tissue itself. It has been recognized that the intercellular network of immunometabolism can be affected by diet. Therefore, research has emerged for foods capable of modulating these responses, which could benefit pathological conditions such as obesity. Current evidence supports the potential of ginger and its bioactive compounds as agents with relevant immunometabolic properties. Preclinical research has shown that ginger is able to modulate key molecular pathways involved in adipogenesis, lipogenesis, lipolysis, fatty acid oxidation, thermogenesis, energy metabolism, and inflammation through mechanisms such as AMPK activation, NF-κB and NLRP3 inhibition, regulation of pro-inflammatory microRNAs (miR-21, miR-132), and induction of changes in the gut microbiota. These effects translate into a reduction in the expression of pro-inflammatory cytokines, including TNF-α, IL-6, and MCP-1; less macrophage infiltration in adipose tissue; and improvements in glycemic control, insulin sensitivity, and serum and liver lipid profiles. In animal studies, supplementation with ginger extracts or isolated compounds has been shown to decrease body weight, reduce adipocyte size, improve glycemia, and attenuate systemic inflammation induced by high-fat diets. Clinical trials report improvements in anthropometric variables and metabolic indicators such as fasting glucose, HbA1c, HOMA-IR, TC, TG, and LDL-C, as well as in inflammatory markers including hs-CRP and IL-6, validating the potential clinical applicability of ginger as a therapeutic complement in the treatment of obesity and pathologies that share immunometabolic dysfunctions.

However, there are some gaps in current knowledge that need to be addressed in future research. Since obesity favors the recruitment of circulating macrophages to adipose tissue and modifies resident macrophages to amplify inflammation, more complex cellular models such as co-cultures with macrophages and adipocytes are needed. In addition, more rigorous in vitro studies are required, integrating the use of positive controls and allowing direct comparisons between active compounds, as well as molecular silencing tools such as CRISPR, which allow us to test the implications of the genes and pathways described here. Furthermore, many studies do not share or evaluate the composition of the extracts they use, which limits the standardization of ginger formulation and dosage and compromises the translational potential of their findings. In vivo studies should also use pharmacological or genetic inhibitors and include pharmacokinetic and bioavailability analyses to corroborate the efficacy and safety of ginger or its derivatives.

It is important to include new experimental approaches in the analysis of immunometabolism [118]. These include flow cytometry for accurate characterization of the diverse cell populations involved, metabolic flux assessment to evaluate cellular metabolism, time-course metabolomic analyses, and transcriptomic tools such as single-cell RNA sequencing and microarrays, which enable the identification of cell- or tissue-specific transcriptional profiles. It would also be valuable to further investigate underexplored mechanisms, such as microbiota modulation, to characterize which microbial species are promoted by prolonged ginger consumption versus isolated compounds, the time required for these changes to occur, their persistence in the gut, and their implications for patients with obesity. Future research is needed to address the mechanisms described for other active components of ginger, accompanied by epigenetic analyses, which would allow the validation of gene regulation mechanisms that could explain the downregulation of specific cytokines and proteins. Also, it is important to use in silico approaches to study immunometabolism, since computational modeling, digital simulations and bioinformatics analysis could complement in vitro and in vivo studies, helping to build more complete immunometabolic networks and models, and would advance the understanding of the functional consequences of the interactions among immune and adipose cells, as well as the identification of new therapeutic targets in obesity and other related pathologies.

This review provides a comprehensive synthesis of the current evidence from in vitro, in vivo, and clinical studies on the immunometabolic effects of ginger in obesity, highlighting consistent findings, proposed mechanisms of action, and relevant research gaps that can guide future studies. It provides a scientific basis for evaluating the therapeutic potential of ginger in metabolic disorders. However, its conclusions are limited by the heterogeneity and methodological variability of the included studies, the predominance of preclinical data, and the lack of standardization in ginger preparations and pharmacokinetic evaluation, which compromises translational applicability.

Overall, ginger is emerging as a promising bioactive agent with consistent immunometabolic effects. While further controlled clinical trials are essential to validate the hypoglycemic, lipid-lowering, antioxidant, immunomodulatory, and antiobesogenic effects of ginger, current evidence suggests that, in the context of obesity, ginger may be very valuable in preventing comorbidities such as DM2, dyslipidemia, and MetS. These findings also emphasize the safety of ginger consumption at the doses studied, as well as its potential to enhance the effects of other lifestyle interventions, such as diet and exercise, in modulating inflammatory and metabolic markers in obese individuals.

## Data Availability

The original contributions presented in this study are included in the article. Further inquiries can be directed to the corresponding author.
